# Reconsidering reviews: the role of scoping reviews in digital medicine and pediatrics

**DOI:** 10.1038/s41746-020-00368-2

**Published:** 2020-12-10

**Authors:** Katherine E. Lewinter, Sharon M. Hudson, Lynn Kysh, Marielena Lara, Cecily L. Betz, Juan Espinoza

**Affiliations:** 1 Division of Pediatric Pulmonology and Sleep Medicine, Department of Pediatrics, Children’s Hospital Los Angeles, Los Angeles, CA USA; 2 Division of General Pediatrics, Department of Pediatrics, Children’s Hospital Los Angeles, Los Angeles, CA USA; 3 Institute for Nursing and Interprofessional Research, Children’s Hospital Los Angeles, Los Angeles, CA USA; 4 RAND Corporation, Santa Monica, CA USA

**Keywords:** Health care, Business and industry, Research data

## Abstract

Digital health is a rapidly developing field which is positioned to transform the manner in which healthcare is delivered, especially amongst adolescents and young adults. In order to assess the efficacy of novel medical devices, clinicians and researchers often turn to the literature for guidance. Randomized control trials and the systematic reviews and meta-analyses that they inform are considered to be at the top of the evidence hierarchy. While they are excellent tools to identify and to summarize the best available evidence to answer a specific research question, they are poorly equipped to provide a more expansive understanding of the body of relevant literature in a timely manner. In this letter we discuss the utility of the scoping review, an underutilized style of academic writing designed to map key concepts in a body of literature. This method is ideal when reporting on the fast-paced field of digital medicine, as it allows for rapid synthesis of the available literature.

Digital medicine is a novel and rapidly evolving field which has the potential to revolutionize the way in which healthcare is delivered^1^. Digital health technology can be utilized for numerous purposes including patient data collection, educational endeavors, improvement of medication compliance, and augmentation of clinician–patient communication. Systematic reviews have evaluated the efficacy of numerous eHealth interventions designed to target various health behaviors in pediatric patients with chronic diseases such as sickle cell disease^2^ and childhood cancer^3^. It has also been suggested that mobile phone apps may offer a feasible mechanism for implementing health interventions with the potential to impact health behaviors^4^. However, more research is required in this area. According to a national survey conducted by Common Sense Media, ~89% of teenagers own smartphones. This percentage has increased significantly since 2012, when only 41% of teens were in possession of these devices^5^. Thus, digital health technologies arguably provide the greatest benefit to adolescents, the population most tied to social media and smart devices, especially as they transition into adulthood and learn to manage their healthcare needs^6^.

In order to assess the efficacy of novel medical devices, clinicians and researchers often turn to the literature for guidance. Randomized control trials (RCTs) and the systematic reviews and meta-analyses (SRMAs) that they inform are considered to be at the top of the evidence hierarchy^7^. However, the rate at which new publications become available is far outpaced by the brisk development of new technologic advancements. In contrast to SRMAs, which are driven by focused primary questions, scoping reviews aim “to map the literature on a particular topic or research area and provide an opportunity to identify key concepts; gaps in the research; and types and sources of evidence to inform practice, policymaking, and research”^8^. Scoping reviews include diverse study types, presentations, and publications; this breadth and flexibility is their strength, and may be the best evidence assessment approach for the fast-moving field of digital medicine.

To illustrate, an RCT funded by a R01 grant takes ~3–5 years to plan and implement. Thus, by the time it is published and subsequently included in a SRMA, 10 or more years may have passed since the original research question was posed. In that same period of time, most digital technologies will have likely undergone one or possibly two significant revolutions as hardware and software are often upgraded on a yearly basis^9^. The findings of a 10-year-old study may be irrelevant to the currently available technology and clinical practice. Thus, the natural academic and funding cycle that governs the timelines of RCTs and SRMAs may lead to findings reported in manuscripts that are obsolete even before they are published.

Well-designed RCTs and SRMAs rely on the Preferred Reporting Items for Systematic Reviews and Meta-Analysis (PRISMA), Methodological Expectations of Campbell Collaboration Intervention Reviews (MECCIR), Methodological Expectations of Cochrane Intervention Reviews (MECIR), and other protocols that are highly structured and prescribed in order to identify and to summarize the best quality evidence for a specific research question^10^. This necessarily strict and rigorous process limits SRMAs from providing a more expansive understanding of the body of relevant literature, thereby hindering timely contributions to the state of the science which are needed for cutting-edge application. SRMAs are also not designed to provide insights regarding clinical questions that cannot be easily addressed by RCTs or other more rigorously controlled studies. Entire segments of the literature which contribute to the development of a field of inquiry, such as cross-sectional and qualitative designed investigations, are not included as they are deemed less rigorous.

This plight of outdated and potentially irrelevant publications pertaining to digital medicine and technology is even more complicated in pediatrics, a field which is grossly underfunded as a whole. According to the 2015 US Census Bureau Data, individuals <18 years of age represented nearly one-quarter of the US population. However, of the $30 billion NIH budget that year, the total NIH pediatric research portfolio was only $3.6 billion, ~12%^11^. The Eunice Kennedy Shriver National Institute of Child Health and Human Development (NICHD) received $1.3 billion, <5%. This is not a new problem. As early as the mid-1990s, the US congress acknowledged that inadequate resources and attention were devoted to pediatric research conducted and supported by the NIH^12^. Recent data demonstrates that the static allocation of NIH funding for pediatric research coupled with reductions in the purchasing power of budgetary funding is negatively affecting the advancement of pediatric science^11,13^. The NIH is not unique in this inequity. It also pervades industry, academia, and other governmental agencies. This lack of funding and prioritization leads to reduced support for pediatric researchers, fewer high-quality RCTs, and even fewer SRMAs. The downstream effect is the publication of pediatric systematic reviews which are often only able to identify a handful of studies, leading to the ever-too-common statement “there is insufficient evidence to form a conclusion”^14^.

Our Pediatric Asthma and Digital Health Research Group, which includes a research librarian, conducted searches on PubMed consisting of Medical Subject Headings (MeSH) terms and Title/Abstract keywords that combined the concept of pediatrics with systematic review, meta-analysis, or scoping reviews (Table 1). Variations in spelling and pluralization were included in order to effectively create a collection of citations that represent the publication of these methods in medicine. Our exploratory analysis showed that as of 2020, 24% of all published SRMAs were in pediatrics. Similarly, 25% of published scoping reviews addressed pediatric topics. Overall, SRMAs outnumber scoping reviews by a ratio of 57:1. Within pediatric topics, scoping reviews are outnumbered by SRMAs by a similar proportion, while being an order of magnitude fewer than adult studies. Taken together, these numbers demonstrate the significant underrepresentation of a pediatric focus among all SRMAs and scoping reviews.

**Table 1:** PubMed search strategies.

Search name	Search syntax	*N* of records
All systematic reviews and meta-analyses	(“Systematic Review” [Publication Type] OR (systematic[sb]) OR Meta-Analysis[Publication Type] OR “systematic review”[Title] OR “systematic reviews”[Title] OR meta-analysis[Title] OR metaanalysis[Title] OR metanalysis[Title] OR meta-analyses[Title] OR metaanalyses[Title] OR metanalyses[Title] OR PRISMA[Title])	239,473
All scoping reviews	“scoping review”[Title]	4187
Pediatric systematic reviews and meta-analyses	(“Infant”[Mesh] OR “Child”[Mesh] OR “Adolescent”[Mesh] OR “Minors”[Mesh] OR “Pediatrics”[Mesh] OR “Pediatricians”[Mesh] OR “Hospitals, Pediatric”[Mesh] OR neonat* OR newborn OR newborns OR infan* OR baby OR babies OR nursery OR nurseries OR toddler OR toddlers OR preschool* OR pre-school* OR kindergarten* OR kid OR kids OR juvenile OR juveniles OR youth OR youths OR youngster OR youngsters OR girl OR girls OR boy OR boys OR preadolescen* OR pre-adolescen* OR preteen* OR adolescen* OR teen* OR pediatric*) AND (“Systematic Review” [Publication Type] OR (systematic[sb]) OR Meta-Analysis[Publication Type] OR “systematic review”[Title] OR “systematic reviews”[Title] OR meta-analysis[Title] OR metaanalysis[Title] OR metanalysis[Title] OR meta-analyses[Title] OR metaanalyses[Title] OR metanalyses[Title] OR PRISMA[Title])	57,282
Pediatric scoping reviews	(“Infant”[Mesh] OR “Child”[Mesh] OR “Adolescent”[Mesh] OR “Minors”[Mesh] OR “Pediatrics”[Mesh] OR “Pediatricians”[Mesh] OR “Hospitals, Pediatric”[Mesh] OR neonat* OR newborn OR newborns OR infan* OR baby OR babies OR nursery OR nurseries OR toddler OR toddlers OR preschool* OR pre-school* OR kindergarten* OR kid OR kids OR juvenile OR juveniles OR youth OR youths OR youngster OR youngsters OR girl OR girls OR boy OR boys OR preadolescen* OR pre-adolescen* OR preteen* OR adolescen* OR teen* OR pediatric*) AND “scoping review”[Title]	1031

The dramatic increase in the number of scoping reviews in medicine (Fig. 1) is likely secondary to their utility and timeliness. Scoping reviews are an attractive scientific alternative and may be a better tool than SRMAs for understanding the range and level of evidence in pediatric topics^15,16^. In particular, scoping reviews can be very useful in the assessment of digital health as the academic and funding life cycles of RCTs and SRMAs limit their ability to address the need for timely synthesis of the evidence to allow for an impact on such a quickly evolving field. For example, a recent scoping review of digital health apps in inflammatory bowel disease by Yin et al provides a systematic overview of apps that have been clinically evaluated, a summary of the evidence from each study, key features, and emerging themes and trends in the field^17^. The more inclusive nature of scoping reviews allowed the authors to broadly group apps into categories (education, monitoring, treatment, follow-up, and patient satisfaction), to determine how they related to each other, and to make observations and recommendations about gaps in knowledge and future opportunities for research.

**Fig. 1:**
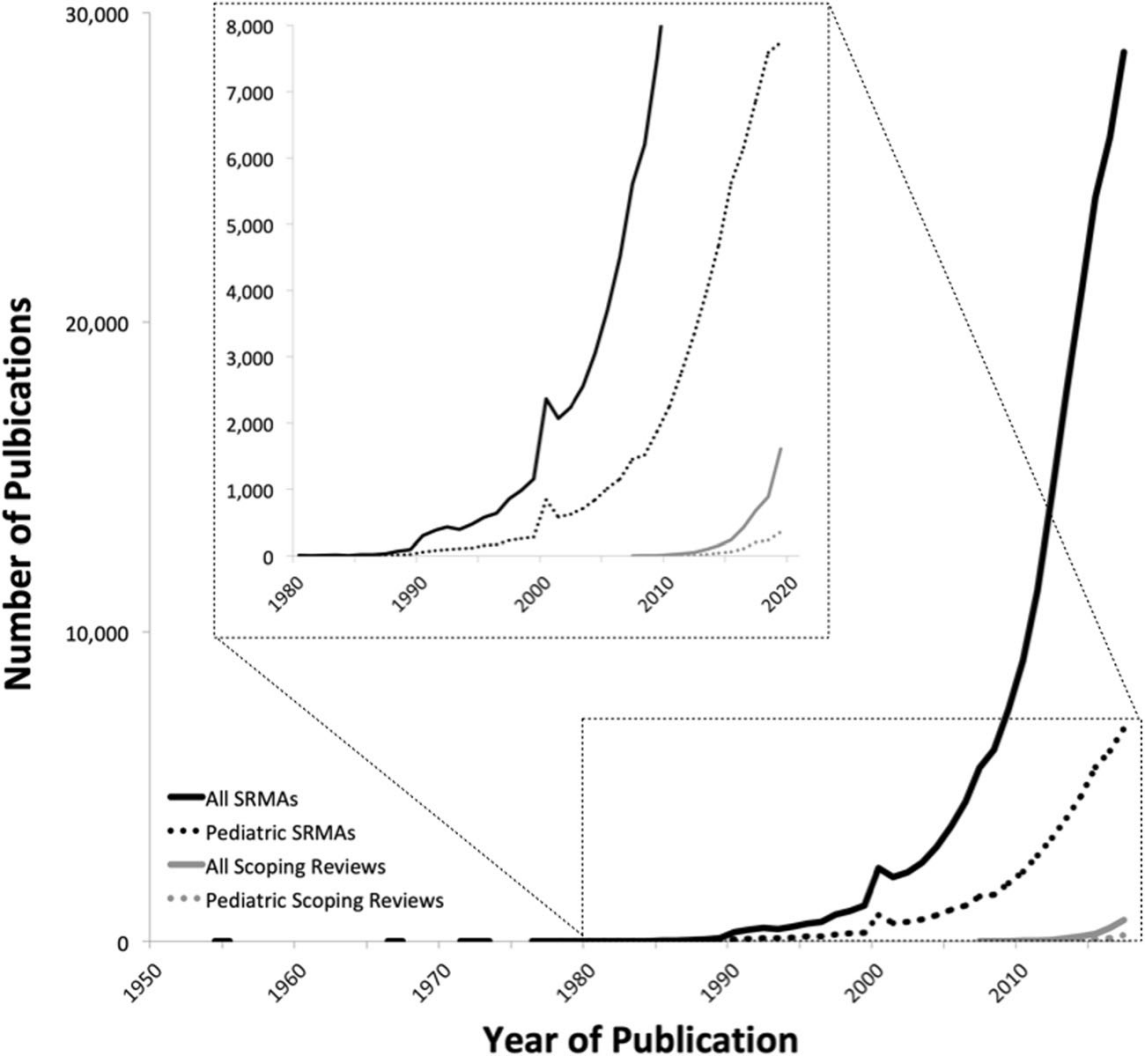
Rate of publication of reviews over time. Graphical representation of the rapid increase in scoping reviews publication. SRMA systematic reviews and meta-analyses.

Despite the utility of scoping reviews, a negative bias exists as they are thought to be less rigorous and less structured than traditional SRMA^18^. The implementation of standardized reporting guidelines for scoping reviews is certainly imperative to ensuring consistency in the literature. The high-impact pediatric journals (*Pediatrics*, *JAMA Pediatrics*, *Journal of Pediatrics*, *Child Development*, *The Journal of Child Psychology and Psychiatry*) do not include scoping reviews as article submission options in their author guidelines, even while specifically identifying systematic reviews, meta-analyses, and narrative reviews as acceptable formats. This academic bias is likely to make scoping reviews less attractive projects for pediatric researchers, resulting in missed opportunities to meaningfully contribute to the literature and to advance pediatric research. A well-designed scoping review could potentially offer crucial insight into the implementation of new technological advances designed to monitor various medical conditions, including the impact that these devices have on patient education, quality of life, integration of patient data into the electronic health record, and other pertinent health outcomes^17,19^. In the case of digital health, scoping reviews will become absolutely necessary to better understand the development and implementation of new devices at an appropriate pace. This is perhaps most pressing in pediatrics, a population who is so dependent on technology. We hope that these comments call attention to this important issue.

## Data Availability

This manuscript is a commentary and not an original study. The data discussed herein is freely available from PubMed, but may also be requested from the corresponding author.
